# Nanoporous gold: a hierarchical and multiscale 3D test pattern for characterizing X-ray nano-tomography systems

**DOI:** 10.1107/S1600577518015242

**Published:** 2019-01-01

**Authors:** Emanuel Larsson, Doğa Gürsoy, Francesco De Carlo, Erica Lilleodden, Malte Storm, Fabian Wilde, Kaixiong Hu, Martin Müller, Imke Greving

**Affiliations:** a Institute of Materials Research, Helmholtz-Zentrum Geesthacht, Max-Planck-Straße 1, Geesthacht 21502, Germany; b Advanced Photon Source, Argonne National Laboratory, Argonne, IL 60439, USA; c Department of Electrical Engineering and Computer Science, Northwestern University, Evanston, IL 60208, USA; d Institute of Advanced Ceramics, Hamburg University of Technology, Hamburg 21073, Germany; e Diamond Light Source Ltd, Didcot, UK; f School of Logistics Engineering, Wuhan University of Technology, 1040 Heping Road, Wuhan, Hubei 430063, People’s Republic of China

**Keywords:** full-field transmission X-ray microscopy, TXM, 3D test pattern, nanoporous gold, sample-stage movement, realignment of projections

## Abstract

Full-field transmission X-ray microscopy (TXM) is a well established technique, available at various synchrotron beamlines around the world as well as by laboratory benchtop devices. One of the major TXM challenges, due to its nanometre-scale resolution, is the overall instrument stability during the acquisition of the series of tomographic projections. The ability to correct for vertical and horizontal distortions of each projection image during acquisition is necessary in order to achieve the effective 3D spatial resolution. The effectiveness of such an image alignment is also heavily influenced by the absorption properties and strong contrast of specific features in the scanned sample. Here it is shown that nanoporous gold (NPG) can be used as an ideal 3D test pattern for evaluating and optimizing the performance of a TXM instrument for hard X-rays at a synchrotron beamline. Unique features of NPG, such as hierarchical structures at multiple length scales and high absorbing capabilities, makes it an ideal choice for characterization, which involves a combination of a rapid-alignment algorithm applied on the acquired projections followed by the extraction of a set of both 2D- and 3D-descriptive image parameters. This protocol can be used for comparing the efficiency of TXM instruments at different synchrotron beamlines in the world or benchtop devices, based on a reference library of scanned NPG samples, containing information about the estimated horizontal and vertical alignment values, 2D qualitative parameters and quantitative 3D parameters. The possibility to tailor the ligament sizes of NPG to match the achievable resolution in combination with the high electron density of gold makes NPG an ideal 3D test pattern for evaluating the status and performance of a given synchrotron-based or benchtop-based TXM setup.

## Abstract 2

Nanoporous gold is hereby proposed as an ideal 3D pattern for characterizing the performance of hard X-ray nano-imaging systems. While a wide range of techniques such as ptychography, holography or other forms of nano-probes will benefit from this development, here its utility is demonstrated by characterizing the transmission X-ray microscope at beamline P05, DESY, which is a full-field technique.

## 1. Introduction   

Standardized 2D test patterns for both full-field and scanning hard X-ray microscopy (XRM) such as the Siemens star have existed for many years, although the amount of available 3D test patterns with well characterized complex structures and strong X-ray interaction are still very limited (Holler *et al.*, 2014 ▸). One major challenge in TXM and in nano-imaging systems in general is the overall stability of the setup during a tomographic acquisition of images. At nanometre length scales, imperfections in rotation stage motion (often called runout errors or spindle errors) become more noticeable. While the synchronous or reproducible component of runout errors of rotation stages can be characterized in advance and corrected for, using *e.g.* an interferometer (Stankevič *et al.*, 2017 ▸), this is not the case for asynchronous errors due to imperfect roundness of bearings and other factors.

If the use of online metrology like interferometry or precise capacitive sensors is not an option, one needs to rely on post-processing to correct for these errors. Therefore, in order to achieve the 3D spatial resolution of the effective detector pixel size, the vertical and horizontal shift of each image with respect to a common rotation axis have to be determined and corrected for in the tomographic reconstruction process. There is a class of ‘iterative reprojection’ techniques (Ollinger, 1990 ▸; Duan *et al.*, 2009 ▸) which have been demonstrated to yield a good solution to the alignment problem through a succession of iterative alignment steps following tomographic reconstruction steps.

The effectiveness of such an image correction is also heavily influenced by the absorption properties and sample-specific features of the scanned sample. Often a high signal-to-noise contrast and rich structural features are required as strong markers for the success of alignment algorithms. In this paper we show how high-absorbing nanoporous gold (NPG) can be used as an ideal 3D test pattern for evaluating and optimizing the performance of a TXM instrument, using a combination of rapid-alignment of the acquired projections followed by the extraction of a set of pre-defined 2D- and 3D-descriptive image parameters. Such extracted parameters can also be used for comparing the efficiency of TXM instruments at different synchrotron beamlines around the world (Chu *et al.*, 2008 ▸; De Andrade *et al.*, 2016 ▸; Andrews *et al.*, 2009 ▸) or benchtop devices (Lavery *et al.*, 2014 ▸; Takano *et al.*, 2017 ▸).

Recently NPG has received an increasing amount of interest in the materials science world due to its functionality dictated by its morphological and topological properties (Qi & Weissmüller, 2013 ▸; Lilleodden & Voorhees, 2018 ▸; Weissmüller & Sieradzki, 2018 ▸). Investigations of the 3D network structure of NPG have already been carried out by different research groups using both focused ion-beam scanning electron microscopy (FIB-SEM) tomography (Mangipudi *et al.*, 2016 ▸; Hu *et al.*, 2016 ▸), Zernike phase-contrast full-field TXM at beamline 32-ID at the Advanced Photon Source at Argonne National Laboratory in Chicago, USA (Chen *et al.*, 2010 ▸) and using scanning TXM, more precisely ptychographic X-ray computed tomography (PXCT) followed by a correlation with both FIB-SEM tomography and electron tomography (ET) (Fam *et al.*, 2018 ▸). The FIB-based method allows high spatial resolution while accessing volumes large enough to be considered as representative of the bulk structure. Unfortunately, the destructive character of this method obviates the ability to observe the evolution of its 3D structure, be it through coarsening or deformation. A non-destructive technique for 3D structural characterization such as TXM in this case is critical to addressing many outstanding questions regarding the scientific understanding of this unique material for a variety of applications.

The novelty of this article is the proposed combination of utilizing NPG as a high-absorbing 3D test pattern for hard X-ray TXM and the application of the joint re-projection method (Gürsoy *et al.*, 2017 ▸) followed by both qualitative and quantitative image analyses, which allows an in-depth characterization of the experimental instrument. This combination provides a strong experimental protocol for evaluating the overall status and quality of a given synchrotron-based or benchtop-based TXM instrument and comparing them with each other based on a reference library of scanned NPG samples. Such a library can contain information about the corrected horizontal and vertical shift of the pixels, 2D qualitative parameters such as contrast-to-noise ratio and quantitative 3D parameters such as percentage volume, specific surface area, descriptors of 3D structural symmetry, connectivity density and ligament and pore size distribution. A direct comparison of one TXM instrument with the other becomes possible since the ligaments sizes of NPG can be tailored from the micrometre-to-nanometre range, with respect to the given resolution of the investigated instrument.

## 2. Methods   

### 2.1. Nanoporous gold   

The targeted electrochemical dealloying of Ag-rich AgAu alloys can result in NPG, a bicontinuous network structure that can be coarsened through annealing (Li & Sieradzki, 1992 ▸). Importantly, NPG displays a high specific surface area, high electrical conductivity and good mechanical properties, making it very interesting for sensing, actuating and barrier coating applications (Collinson, 2013 ▸). The structural length-scales (ligament sizes) of NPG can be tailored to different structural thicknesses ranging from 20 nm up to 1 µm, which allow a tailorable mechanical response (Hodge *et al.*, 2007 ▸).

The investigated NPG sample was produced from a millimetre-sized fully dense electrochemically dealloyed Ag_75_Au_25_ alloy (with an average grain size of around 50 µm), followed by annealing for 420 min as described further by Hu *et al.* (2016 ▸). For FIB-SEM tomography, NPG is commonly infiltrated with a second phase such as ep­oxy resin, in order to provide extra support during the FIB process (Peña *et al.*, 2018 ▸), as well as for removing background signal from subsequent layers during the SEM imaging, thereby aiding image segmentation (Sabharwal *et al.*, 2017 ▸). For TXM measurements of NPG, no ep­oxy infiltration step is needed, and the sample can thus be scanned directly in air, which also provides a higher contrast, since the air-to-Au contrast is greater than that of ep­oxy-to-Au.

**Figure 1:**
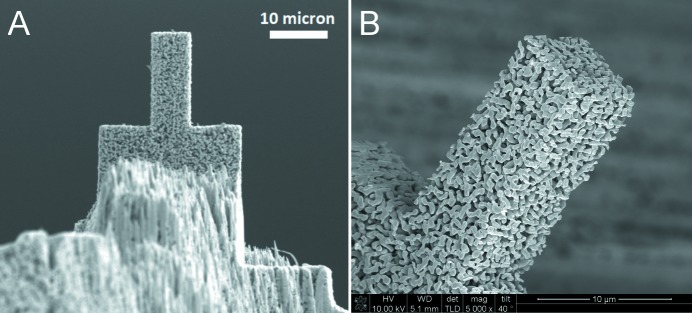
A micropillar with a rectangular cross section fabricated via focused ion beam milling. The base of the pillar is fabricated as a stepped lamellar structure.

For the hereby described TXM measurements a micropillar of NPG with a rectangular cross-sectional area, as shown in Fig. 1 ▸, was prepared using FIB milling, applied to a smaller piece of the bulk NPG specimen. A FEI Nova200 dual beam microscope was used for this purpose, with a 30 kV Ga ion beam. In order to achieve a 360° visibility, as required for TXM, higher currents were used to remove larger volumes of material surrounding the region which would become the micropillar. This leads to a stepped lamellar structure underneath the pillar, as can be seen in Fig. 1(*A*) ▸. Hereinafter, a cleaning cross-section milling protocol at a current of 300 pA was applied to all surfaces of the micropillar. No FIB-related artefacts were observed on the micropillar, as shown in Fig. 1(*B*) ▸. Hereinafter, the NPG sample was cut out and mounted onto a SEM sample holder stub using platinum.

In order to establish non-destructive compression testing of NPG, the sample was initially scanned with the same TXM setup as described under Section 2.3, but this time using a PCO edge 4.2 camera (https://www.pco.de/scmos-cameras/pcoedge-42/) with an exposure time of 10 s, and subsequently compressed to a nominal plastic strain of 12%, followed by further scanning with TXM using a camera with lower exposure times, as described further under Section 2.3. Due to the long exposure time of the initial scan the recorded projection images could not be sufficiently corrected for horizontal and vertical drift. Therefore, only the results of the compressed NPG pillar are hereby reported.

### 2.2. NPG as an ideal 3D test pattern for full-field TXM   

The high-absorbing capabilities of gold (Au) itself together with the ability to tailor the ligament sizes of NPG with respect to the achievable resolution makes NPG an ideal 3D test pattern for evaluating, optimizing and comparing the performances of TXM instruments at different synchrotron beamlines or benchtop devices, based on a set of pre-defined 2D-qualitative and 3D-quantitative parameters. Furthermore, the effectiveness of the image correction algorithm as further described under Section 2.4 is also heavily influenced by the absorption properties and sample-specific features of the scanned sample, which makes NPG with its high-absorbing properties and complex structure an ideal 3D test pattern for this purpose. An optimal 3D test sample should, apart from having a well characterized complex structure and strong X-ray interaction, also be resistant to radiation damage (Holler *et al.*, 2014 ▸). Au in this case does not suffer from severe radiation-induced damage, which is why it is also commonly used as a zone material in X-ray optics (Jefimovs *et al.*, 2008 ▸). Also, for this purpose NPG (as being made up by Au) can thus be considered an optimal 3D test pattern for TXM.

### 2.3. Setup of the full-field transmission X-ray microscopy technique   

The NPG sample (with a size of 8 µm × 8 µm × 14.5 µm) was scanned with a TXM instrument (Ogurreck *et al.*, 2013 ▸) at the P05 imaging beamline (Greving *et al.*, 2014 ▸), operated by the Helmholtz-Zentrum Geesthacht (HZG) at the PETRA III storage ring at the Deutsches Elektronen-Synchrotron (DESY), in Hamburg, Germany. The scanning parameters were the following: energy, 11 keV; exposure time, 1.0 s; number of projections, 450; sample-to-detector distance, 18.8 m; field of view (FOV), 30 µm × 30 µm; effective isotropic pixel size, 19.8 nm. The obtained 2D spatial resolution was 50 nm as defined by the half-period of the spatial frequency, as earlier shown by Greving *et al.* (2017 ▸). The utilized Au Fresnel zone plates (FZPs) and beam-shaping illumination optics (Jefimovs *et al.*, 2008 ▸) were designed and fabricated by Paul Scherrer Institute in Villigen, Switzerland. The characteristics of the utilized beam-shaping illumination optics were the following: zone material, electroplated Au with a structure height of around 1100 nm; support membrane, Si_3_N_4_ with a thickness of 250 nm; mosaic, 50 µm × 50 µm × 50 µm sub-fields; outermost zone width, 70 nm (sub-fields in the corners); size, 1.8 mm × 1.8 mm (36 × 36 sub-fields); chip dimensions, 6 mm × 6 mm. The characteristics of the utilized FZP were the following: zone material, Au; support membrane, Si with a size of 6 mm × 6 mm; outermost zone width, 80 nm; thickness of absorber, >1.4 µm; design energy, 14 keV. Furthermore, a Hamamatsu C12849-101U X-ray sCMOS camera (http://www.hamamatsu.com/eu/en/product/category/5000/5005/C12849-101U/index.html) was used for acquiring the projection images. Ten dark and three flat-field projections were acquired before and after the tomographic scan. In addition, three flat-field projections were acquired at every 15th angular step. The camera has the following characteristics: physical pixel size, 6.5 µm; scintillator, Gadox 10 µm (placed directly on top of the fibre optics of the camera, thereby resulting in a very high photon efficiency); effective number of pixels, 2048 × 2048. A decoherer based on normal paper material was positioned directly at the outlet of the vacuum beam pipe, around 40 cm in front of the condenser ring, to reduce unwanted phase contrast effects.

### 2.4. Preprocessing – rapid alignment of projections   

**Figure 2:**
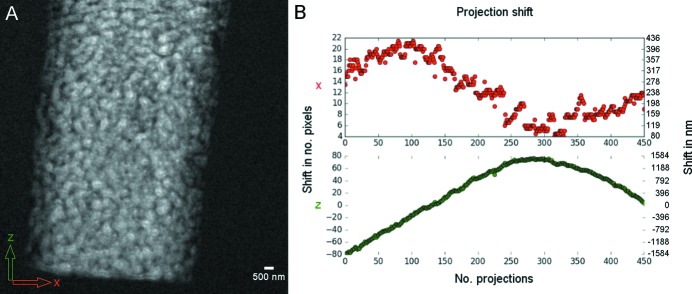
(*A*) Flat-field original projection displayed on a 32-bit grey-level scale. (*B*) Observed shift in number of pixels, and in nm, in the horizontal (*x*) and vertical (*z*) direction. (The effective pixel size is 19.8 nm in all directions *x*, *y* and *z*.)

After acquisition of the projection images the joint re-projection method (Gürsoy *et al.*, 2017 ▸) for simultaneous alignment and reconstruction was applied. This method uses the simultaneous iterative reconstruction technique (SIRT) for performing reconstruction iterations, and cross-correlation for registering the raw projection image with the re-projected image at each iteration, as shown in Fig. 2 ▸. The algorithm runs until convergence is reached. The algorithm is implemented and available publicly in *TomoPy* (Gürsoy *et al.*, 2014 ▸) (https://github.com/tomopy/tomopy). The original acquired projection images are available publicly (Larsson *et al.*, 2018 ▸) via the *TomoBank* repository (De Carlo *et al.*, 2018 ▸) at the following link: https://tomobank.readthedocs.io/en/latest/source/data/docs.data.nano.html#drift.

**Figure 3:**
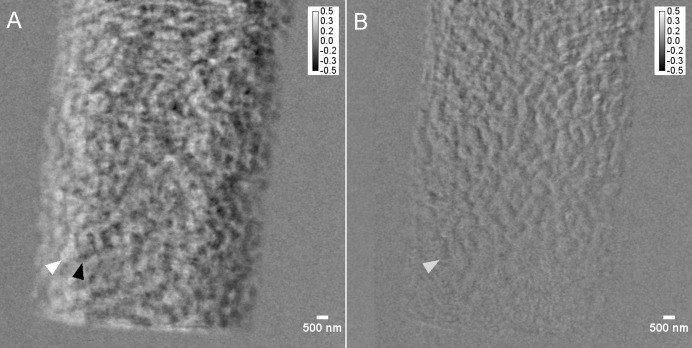
Flat-field-corrected projections of a projection acquired at 0° subtracted with a projection acquired at 180° (after horizontal flipping). (*A*) Original misplacement of sample and (*B*) after correction of the sample movement via rapid alignment and forward projecting (number of iterations: 40), which severely reduces the sample-movement error seen in (*A*), especially at the top of the sample (displayed as upside-down in XRM mode). The images are displayed on a 32-bit grey-level scale. Similar features have been marked with arrow heads. (Note that the correction of the projections worked better on the top of the sample than on the bottom part, which is most likely related to a slight wobble or tilt of the rotation axis, which could not be ‘entirely’ corrected for by the rapid alignment algorithm.)

The accuracy of the applied alignment is revealed in Fig. 3 ▸, which presents the resulting image of a subtraction of a projection acquired at 0° of rotation with one at 180° (after ‘horizontal flipping’), with the pre-defined condition that the resulting image should be ‘empty’, following the idea that the sample should return to its original position if no sample drift or vibrations are present.

**Figure 4:**
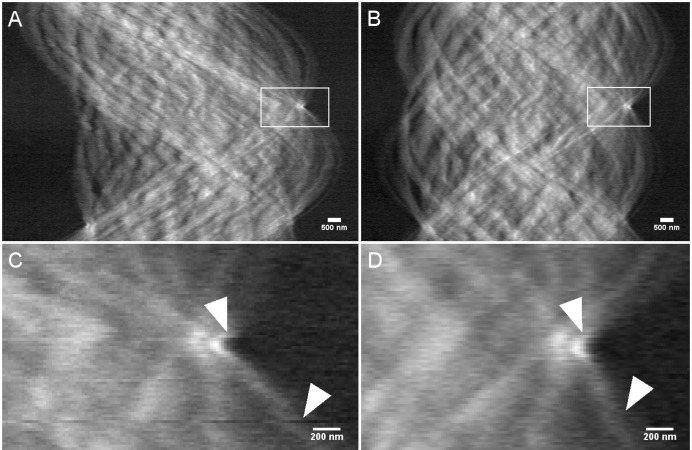
Sinograms of NPG. (*A*) Original and (*B*) after rapid alignment and forward projection (number of iterations: 40). Zoomed-in views of (*A*) and (*B*) are shown in (*C*) and (*D*), respectively, where the arrows point at well-corrected sample features, which improve the continuity of the sinogram. The images are displayed on a 32-bit grey-level scale. [Note that the rapid alignment shifts the projection images also in the *z*-direction, thus the data shown in the sinogram of row *i* in (*A*) and (*C*) will be different from that after registration, as shown in (*B*) and (*D*). Hence, the ‘exact’ sinogram *i* no longer exists, and instead a slightly modified sinogram has been created. However, key features in the non-aligned and aligned sinograms can still be traced for comparison as highlighted by the white arrow heads.]

An improvement can also be seen directly in the resulting sinogram, where a much smoother sinogram is obtained after applying rapid alignment, as seen in Fig. 4 ▸, thereby leading to an improved reconstruction, with fewer streak-artefacts.

**Figure 5:**
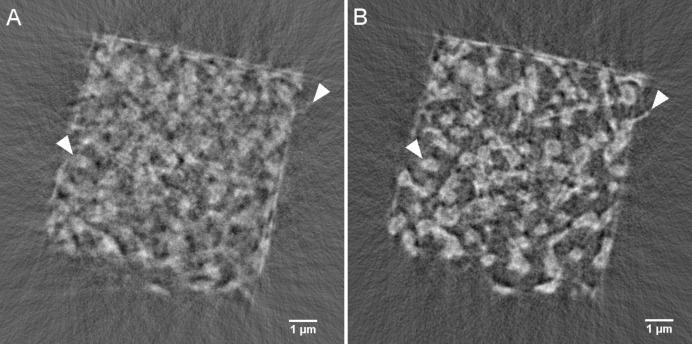
Reconstructed slices. (*A*) Original and (*B*) after rapid alignment of the projections followed by reconstruction with forward projection (number of iterations: 40). A much more accurate reconstruction with fewer streak artefacts can be seen in (*B*) in comparison with (*A*). Arrow heads highlight an improved reconstruction of sample-specific features and edges. The images are displayed on a 32-bit grey-level scale.

Hereinafter, the linear X-ray attenuation coefficient (LAC) [cm^−1^] was reconstructed on a 32-bit grey-level scale using *TomoPy* and an iterative reconstruction algorithm (Gürsoy *et al.*, 2014 ▸) based on ‘forward projection’ (number of iterations, 40). A comparison of an original slice with a slice where prior rapid alignment of the projections had been applied (hereinafter referred to as ‘aligned slice’) is shown in Fig. 5 ▸.

### 2.5. Qualitative 2D image analysis   

After reconstruction, the contrast-to-noise ratio (CNR) between the Au and air was measured in ten different slices (original 32-bit grey-level scale) using equation (1), as proposed by Muhogora *et al.* (2008 ▸), where *I* is the intensity and σ is the standard deviation of the quantified grey-level values inside a considered circular region of interest (area, 0.08 µm^2^) of either Au or air,
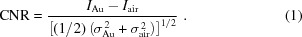
This equation has also been proven to be efficient for evaluating the CNR of both absorption- and phase-contrast-based tomographic data sets obtained by synchrotron microtomography (SRµCT), as shown by Mohammadi *et al.* (2014 ▸).

### 2.6. Qualitative estimation of the 3D resolution   

The 3D resolution was estimated via Fourier shell correlation (FSC) as defined by the half-period of the spatial frequency (Van Heel & Schatz, 2005 ▸) on the largest sub-volume which could be extracted without having to rotate the sample. Hence, an individual volume of interest (VOI) size of 4.0 µm × 4.0 µm × 12.7 µm was selected far away from the sample edges. The FSC was estimated via sequential comparison of the slices, using the ‘EM2EM/FSC’ functions of the *IMAGIC* image processing package (Image Science Software GmbH), which is available as a ‘free download’ at the following link (https://download.imagescience.de/index.html#em2em_fsc_download). The following computational parameters were used when estimating the 3D resolution by FSC: threshold for FSC curve (sigma), 3.0; point-group symmetry, C1; filling degree, 0.66. Since the 3D resolution was estimated on individual slices via sequential comparison, rather than their average, as further addressed by Holler *et al.* (2014 ▸), only the lowest obtainable 3D resolution was reported in this article.

### 2.7. Post-processing – image processing of reconstructed slices   

After reconstruction, slices were down-converted to an 8-bit grey-level scale using *Fiji* (Schindelin *et al.*, 2012 ▸) and subsequently analyzed further using the *Pore3D* software package (Brun *et al.*, 2010 ▸). *Pore3D* is a software package for quantitative analysis of porous media (developed by the SYRMEP group at the Elettra Synchrotron in Trieste, Italy), which only supports 8-bit or 16-bit images, as further described under Section 2.8. The down-conversion did not hamper further image segmentation procedures. The histogram, as shown in Fig. S2 of the supporting information, shows two main peaks associated with air (weakly absorbing part) and Au (strongly absorbing part). The two peaks were separated using standard grey-level thresholding in *Fiji* (Schindelin *et al.*, 2012 ▸), following the application of a 3D mean filter (core: 3 × 3 × 3 pixels) (Ollion *et al.*, 2013 ▸), in order to remove image noise and facilitate the above-mentioned image segmentation, as shown in Fig S3.

### 2.8. Quantitative 3D image analysis   

**Figure 6:**
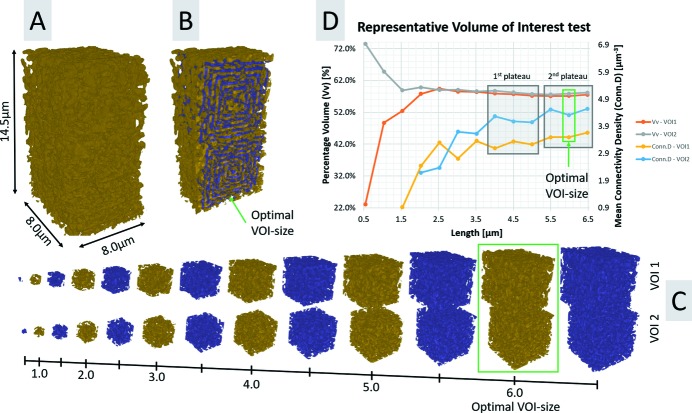
Test for finding the representative volume of interest (RVI-test), based on the Vv as the evaluation parameter and Conn.D as the control parameter for the air part. The optimal VOI size is found when the variation of the Vv and Conn.D are no longer affected by the varying size of the considered VOI. For the considered sample this occurred at 6 µm, at which length scale both the Vv and Conn.D have levelled out and reached a plateau, *i.e.* at the second plateau for the Conn.D. Note that no Conn.D values are provided for VOIs below 1.5 and 2.0 µm for VOI1 and VOI2, respectively, since the VOIs were too small for extracting an image skeleton, with nodes and branches.

Quantitative image analysis requires the selection and analysis of a set of VOIs which are extracted from different positions throughout the sample, at a distance 1 µm away from the sample borders in order to avoid any potential FIB-related artefacts effecting the analysis results. In order to be able to select as large VOIs as possible the slices were rotated 16° counter-clockwise so that the sample edges were aligned with the edges of the image. However, prior to this step, the optimal size of the given VOIs needs to be determined by a test of representative volume of interest (RVI-test) (Bear, 2013 ▸). The optimal VOI size is found when the variation of the percentage volume (Vv) (%) as the evaluation parameter and connectivity density (Conn.D or β) as the control parameter, as further described below, are no longer affected by the varying size of the considered VOI. For the given NPG volume, based on the investigation of two sub-volumes, the RVI-test found a representative VOI size of 6.0 µm × 6.0 µm × 6.0 µm, which was chosen when both the Vv and Conn.D have levelled out and reached a plateau, *i.e.* at the second plateau for the Conn.D, as the maximum mean value and minimum standard deviation of the Conn.D was found here, as shown in Fig. 6 ▸. The result of this test is also in accordance with an RVI-test performed on a FIB-SEM tomography data set of NPG (using the same annealing time), involving in total six sub-volumes (Hu *et al.*, 2016 ▸). Furthermore, choosing the same VOI size for both the TXM data set as for the previously evaluated FIB-SEM data set will also allow a fairer comparison in terms of correlative imaging.

The chosen VOIs were then further analyzed based on a set of morphological structure parameters such as Vv and specific surface area (Sv) (µm^−1^) of both air and Au. All the above-mentioned quantitative analysis was performed using the *Pore3D* software package (Brun *et al.*, 2010 ▸), which is available open source (https://github.com/ElettraSciComp/Pore3D).

The VOIs were also investigated by means of anisotropic measures, such as (1) isotropy and (2) elongation, which are descriptors of 3D structural symmetry. For the isotropy index, a value close to 1 indicates that there is ‘no preferential direction’, whereas a value closer to 0 indicates there is ‘a preferred direction’. Given the latter, the elongation index quantifies the structural symmetry orthogonal to the preferred direction, as quantified by the isotropy index. For the elongation index, a value close to 1 indicates that there is ‘a preferred direction’, whereas a value close to 0 indicates that there is ‘no preferred direction’.

**Figure 7:**
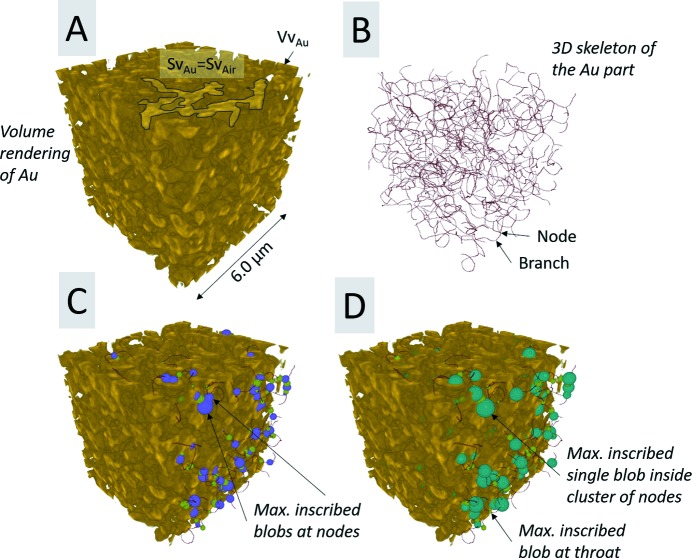
3D rendering of a VOI of (*A*) Au (in golden yellow), (*B*) 3D skeleton (in red) also known as medial axis of the Au part, (*C*) maximal inscribed blobs at ligament nodes (purple) and at ligament throats (light green) and (*D*) maximal inscribed single blob inside a cluster of nodes (maximum node ligament size) (dark green).

Moreover, image skeletons, representing the shortest interconnecting paths throughout both the porous (air) part and Au part of the sample, as shown in Figs. 7(*B*) ▸ and 8(*B*) ▸, were computed using the Gradient Vector Flow (GVF) algorithm (Brun & Dreossi, 2010 ▸) using the following parameters: scale = 0.40, hierarchy = 0.10 and connectivity = 26. Furthermore, Conn.D, which provides a value for how well connected the porous space is, was calculated based on the following equation, β = 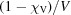, where 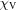 is the Euler number, defined as 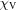 = number of nodes − number of branches for the extracted image skeleton and *V* is the volume (in µm^−3^) of the given VOI. For the given analysis, only the longest connected image skeleton was considered and isolated non-connected branches were ignored.

**Figure 8:**
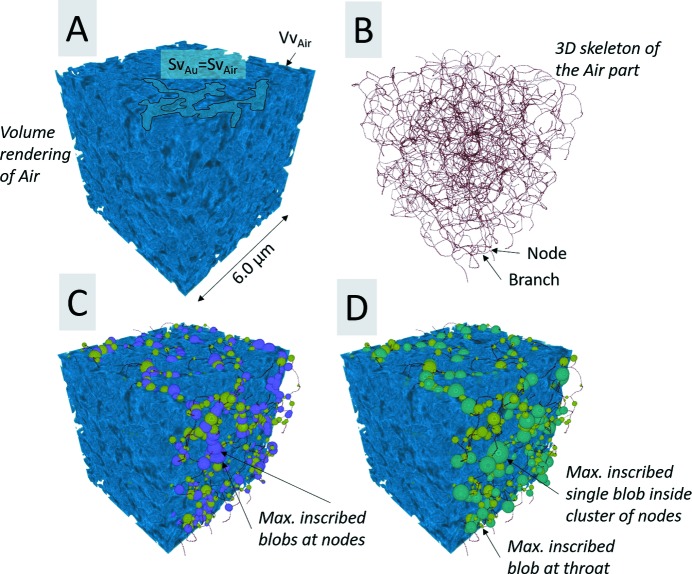
3D rendering of a VOI of (*A*) air (in blue), (*B*) 3D skeleton (in red) also known as medial axis of the air part, (*C*) maximal inscribed blobs at the nodes (purple) and at throats (light green) in the porous space and (*D*) maximal inscribed single blob inside a cluster of nodes (maximum node pore size), *i.e.* at the centre of a large pore (dark green).

Moreover, virtual spheres or ellipsoids, also known as blobs, were inscribed at (1) the nodes and (2) the throats (*i.e.* the maximum opening size between two nodes along a connecting branch on the image skeleton) for both the porous and solid sample part and were allowed to grow until they touched the interface between the two sample parts. Hereinafter, ‘blob analysis’ was applied on the inscribed blobs in order to quantify the node and throat size distribution of the solid part, as shown in Fig. 7(*C*) ▸, and for the porous part, as shown in Fig. 8(*C*) ▸. Furthermore, additional blobs were inscribed inside the cluster of the nodes (hereinafter referred to in general as ‘maximum node size’), in order to quantify the maximal pore size distribution of the porous part, as shown in Fig. 7(*D*) ▸, and the maximal node ligament size distribution of the solid part, as shown and Fig. 8(*D*) ▸. Finally, the sphericity was also estimated for all inscribed blobs.

### 2.9. 3D-rendering   

All the 3D volume renderings of both the entire sample and extracted VOIs were performed using the *VG Studio MAX 3.1* software (Volume Graphics GmbH, Heidelberg, Germany, 2018).

### 2.10. Setting up a public TXM data repository of scanned and analyzed NPG samples will allow a comparison of alignment and characterization techniques   

The hereby proposed ‘experimental protocol’ for (i) recording and correcting the horizontal and vertical shift of the pixels in the projection images, using *e.g.* ‘rapid alignment’ or other available alignment algorithms (Guizar-Sicairos *et al.*, 2011 ▸, 2015 ▸) tested on tomographic phase projections obtained by ptychographic coherent diffractive imaging (CDI), also referred to as scanning X-ray diffraction microscopy, followed by (ii) 2D qualitative evaluation using CNR, (iii) qualitative evaluation of the 3D resolution via FSC (Van Heel & Schatz, 2005 ▸) and (iv) quantitative 3D analysis, based on *e.g.* Vv, Sv, 3D structural symmetry descriptors (iso­tropy, elongation), Conn.D and ligament and pore size distribution, can be used for setting up a public TXM data repository of scanned and analyzed NPG samples (https://tomobank.readthedocs.io/en/latest/source/data/docs.data.nano.html#drift) (Larsson *et al.*, 2018 ▸) at various synchrotron-based or benchtop-based TXM instruments.

Moreover, the performance and quality of the reconstructed images depend both on the instrument itself as well as the chosen alignment algorithm. Therefore, a public TXM data repository will thus allow for the testing and optimization of post-acquisition computational workflows, with respect to the specific instrument, as there is so far no universal alignment algorithm that is optimal for correcting projection images from all types of tomographic instruments.

### 2.11. Setting up a physical library of NPG samples and/or establishing a common process protocol for synthesizing NPG samples on-site will allow a comparison of the efficiency and performance of TXM instruments   

The possibility to tailor the ligament sizes of NPG to match the achievable resolution will allow for a fair comparison for evaluating the efficiency and performance of given TXM instruments. In more detail, NPG samples can also be synthesized for different synchrotron-based and benchtop-based tomography systems, *e.g.* micro- and nano-tomography (including both full-field and scanning TXM, *i.e.* PXCT), with various resolutions. Based on the feedback from the TXM community, as well as the tomography community in general, either (i) a physical library of NPG samples with applicable ligament sizes for various tomography systems can be set up or (ii) a common processes protocol for synthesizing NPG samples with applicable ligament sizes on-site can be established. Such approaches will thus allow for a more direct comparison of the efficiency and performance of tomography instruments with similar resolution.

## 3. Results   

**Figure 9:**
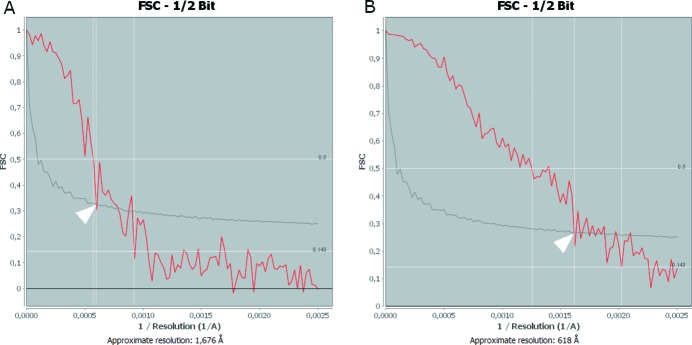
Estimation of the 3D-resolution via FSC sequential comparison, defined by the half-period of the spatial frequency of reconstructed slices. (*A*) Original (167.6 nm) and (*B*) after rapid alignment of the projections (61.8 nm), as indicated by the arrow heads.

**Figure 10:**
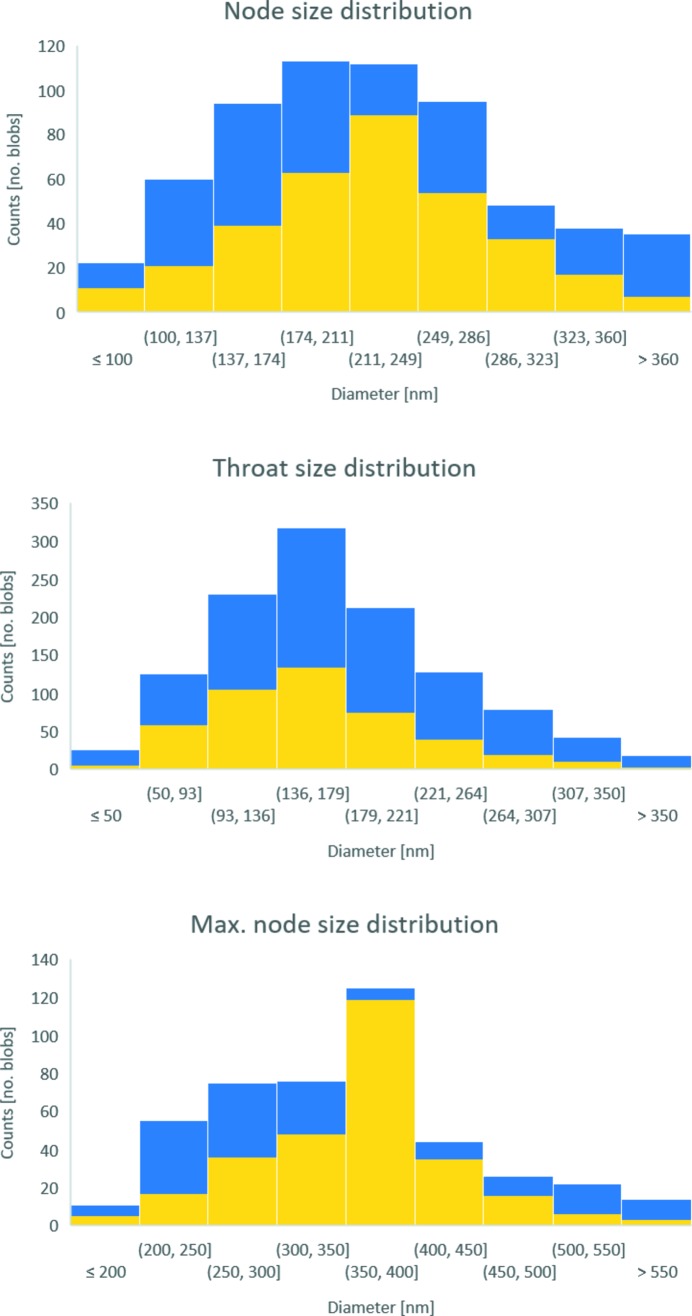
Node, throat and maximum node size distribution of both the air (porous, blue) and Au (solid, yellow) part.

The aligned slice Fig. 5(*B*) ▸ shows a much more accurate delineation of both sample edges and features, compared with the original reconstructed slice, as shown in Fig. 5 ▸. The qualitative 2D analysis also showed that the alignment process allowed an increase of the CNR from 6.35 ± 0.91 (original slice) to 8.28 ± 1.81 (aligned slice), which represents an increase of 30.4%. The qualitative estimation of the 3D-resolution via FSC, as shown in Fig. 9 ▸, reveals that the alignment process increased the 3D resolution by a factor of 2.7, from 168 nm (original slices) up to 62 nm (aligned slices), which corresponds to an increase of about 170% in 3D-resolution. The quantified 3D image parameters shown in Table 1 ▸ reveal that the porous part (air) makes up the bigger part of the considered VOIs, as seen by the quantified Vv. The Sv, on the other hand, is the same for both the porous (air) and solid (Au) part, as expected for a two-component material such as NPG filled with air. The anisotropic analysis reveals a sample with ‘close to’ no preferred orientation in neither direction or plane, with an isotropy index close to 1 and an elongation index close to 0, for both the porous and solid part. The Conn.D based on the number of nodes and branches of the extracted skeletons shows that the porous part is more well connected than the solid part, *i.e.* the ligaments of the NPG. Consequently, the number of inscribed blobs at the nodes and throats (at the branches of the skeleton) and the number of maximal inscribed blobs inside the cluster of nodes is the highest for the porous part, as shown in Fig. 10 ▸. Furthermore, all the considered inscribed blobs presented very high sphericities, with mean sphericity values between 0.90 and 1.00, as shown in Table 1 ▸. Moreover, the node and maximum node size distributions tend to follow a normal distribution, although the throat size distributions tend to follow a slight left-skewed normal distribution for both the porous and solid part, as shown in Fig. 10 ▸. The mean size difference of the inscribed blobs for the porous and solid part, as shown in Table 1 ▸, only differentiated by roughly 5–13 nm, which is below the effective pixel size of 19.8 nm.

## 4. Discussion   

The amount of available 3D test patterns for hard X-ray nano-tomography systems, *e.g.* PXCT and TXM, are still very limited. Holler *et al.* developed an ideal and radiation tolerant 3D test pattern for high-resolution PXCT using a nanoporous SiO_2_ glass (hereinafter referred to as NP-SiO_2_) structure (mean pore size 139 nm) and coated with a Ta_2_O_5_ film (37 nm in thickness) based on atomic layer deposition, which was used for characterizing the isotropic 3D resolution to 16 nm, using FSC at an energy of 6.2 keV (Holler *et al.*, 2014 ▸).

Another potential 3D test pattern for hard X-ray nano-tomography systems, in this case TXM, as shown in this article, is NPG. Only a small amount of studies have so far been reported, where NPG (from a strictly materials science point of view) has been scanned with TXM instruments at synchrotron beamlines (Chen *et al.*, 2010 ▸; Chen-Wiegart *et al.*, 2012 ▸).

One advantage of NPG is that the sample preparation is well established and reproducible (Sun *et al.*, 2014 ▸) as well as less advanced and time-consuming than for NP-SiO_2_, since NPG does not require any coating step. Moreover, NPG is made up of a solid and high-absorbing material structure, whereas NP-SiO_2_ is made up of a hollow SiO_2_ glass structure coated with high-absorbing Ta_2_O_5_ film. Therefore, it can be argued that NPG represents a more stable and robust 3D test pattern, which can thus be more safely transported in between comparable TXM instruments over the world. The higher absorption capabilities of NPG due to its solid structure will also allow a more accurate recording and correction of any potential horizontal or vertical drift by the ‘rapid alignment’ algorithm. Consequently, NPG can be proposed as an optimal 3D test pattern for hard X-ray nano-tomography systems. Since NPG offers a broad range of ligament size distributions from 20 nm up to 1 µm with respect to the achievable spatial resolution, it can also be proposed as a potential ideal 3D test pattern for both micro- and nano-tomography (including both full-field and scanning TXM, *i.e.* PXCT, as well as fluorescence and point projection microscopy).

In this study an NPG sample was used as an ideal 3D test pattern to quantify the performance of the TXM instrument at the P05 beamline at DESY. Selected 3D parameters of the hereby presented TXM data set were directly compared with corresponding ones as quantified by FIB-SEM tomography (with an effective voxel size of 25 nm × 25 nm × 20 nm) for an NPG sample prepared at the same annealing time as one of our earlier studies (Hu *et al.*, 2016 ▸). More precisely, the solid part of the TXM data set presented a Vv of 42.6%, whereas the FIB-SEM data set showed a Vv of 33.4%, which represents a difference of 9.2 percentage points, which could be explained by the prior compression test of 12% plastic strain.

Furthermore, the FIB-SEM data set confirmed a mean ligament size of 420 ± 23 nm, whereas the corresponding parameter from the TXM data set gave a mean maximum node ligament size of 352 ± 95 nm, which represents a difference of 68 nm. This is, however, below the standard deviation of this parameter for the TXM data set and might be directly linked to differences in resolution between the two techniques. Of course, the plastic deformation itself will lead to changes in the average diameter and diameter distribution; it is unclear to what extent pinch-off events during local ligament failure could reduce the average size. In fact, sample compressing studies are of high interest and underscore the importance of such non-destructive techniques for quantifying the 3D structure of NPG. Minor variations can also be related to differences in the sample preparation, where FIB-SEM tomography requires the immersion of NPG in ep­oxy, while TXM has no such requirement.

In a very recent study NPG (with an unknown annealing time) was scanned using both PXCT (effective pixel size, 13.3 nm; isotropic 3D resolution, 23 nm; energy, 8.7 keV) and FIB-SEM tomography (effective pixel size, 12.8 nm) (Fam *et al.*, 2018 ▸). The solid part of the PXCT and FIB-SEM data sets of NPG made up Vv values of 40% and 61%, respectively, which represents a difference of 21 percentage points. In addition, the mean ligament sizes were 211 ± 23 nm and 277 ± 15 nm for the PXCT and FIB-SEM data sets, respectively, which represents a difference of 66 nm, which is much higher than the standard deviation for both techniques.

The fact that the hereby compared 3D quantitative parameters for both the FIB-SEM and TXM data sets are deviating in similar ranges as those quantified by PXCT and FIB-SEM confirms that the investigated NPG sample can be seen as an ideal and representative 3D test pattern for evaluating and improving the status of a TXM instrument for hard X-rays, particularly in combination with the application of the joint re-projection, thereby detecting both sample drift and vibration of the rotation stage. After maintenance of the TXM instrument at the P05 beamline the error of the rotation stage has been reduced significantly, which has drastically improved the accuracy and therefore also the quality of reconstructed images also of other samples scanned at the nano-tomography station at this beamline.

Furthermore, to the best of the authors’ knowledge, this article presents the very first results of the inter-connectivity of both the solid and porous part of NPG, as quantified by full-field TXM. The inter-connectivity of the Au part is 58% lower than that obtained from the FIB-SEM data set (Hu *et al.*, 2016 ▸), for similar NPG samples prepared with the same annealing time. The major reason behind these differences is most likely associated with the implementation of the applied skeletonization algorithms, whereas the utilized GVF skeletonization algorithm (Brun & Dreossi, 2010 ▸), applied for the TXM data set, provides a skeleton with smoothly curved node-to-node and node-to-end branches, also referred to as ’dangling branches’ (Hu *et al.*, 2016 ▸); meanwhile the ‘Skeletonize3D’ algorithm (Lee *et al.*, 1994 ▸), applied for the FIB-SEM data set, provides a skeleton with straight node-to-node branches, followed by the removal of nodes and branches associated with dangling branches, thus creating a skeleton with a fully connected ring structure. The higher inter-connectivity of the Au part of NPG for the FIB-SEM data set can thus be explained by the removal of nodes and branches associated with dangling branches, which leads to an increase in connectivity, according to the formula β = 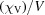 and discrete mathematics, where the number of nodes (subtracted part) will always be greater than the number of branches (added part). If both the total number of nodes and branches are reduced, the connectivity should consequently increase. It also needs to be taken into account that there is no precise general definition of the image skeleton in a 3D volume (Cornea *et al.*, 2007 ▸), where the effectiveness of the skeleton analysis is thus limited by the accuracy of the applied skeleton algorithm (Brun *et al.*, 2010 ▸), as well as by the contrast, resolution and quality of the quantified data set.

Moreover, the final 3D-resolution is primarily limited by the utilized X-ray optics, and secondly by the efficiency of the utilized alignment algorithms, as shown in this article. For the NP-SiO_2_ sample scanned with PXCT at a total dose of 1.2 × 10^10^ Gy (per an entire scan of 720 projections was reported), and beam damage could also be observed, which further limited the final 3D-resolution (Holler *et al.*, 2014 ▸).

On the other hand, the NPG sample scanned by PXCT reported no beam damage, even for a total dose of 8.28 × 10^8^ Gy (per an entire scan of 900 projections) (Fam *et al.*, 2018 ▸). This further supports the use of NPG as an optimal 3D test pattern for nano-tomography systems, *i.e.* PXCT and TXM, and can be incorporated into a physical library of NPG samples.

In order to allow a fair comparison of the performance of various synchrotron-based and benchtop-based TXM instruments, the hereby presented NPG data set has been made available publicly via a proposed TXM library, which can hence be used for further investigations, *e.g.* the calculation of the interfacial shape distribution (Chen *et al.*, 2010 ▸), as well as allowing for investigations using other available or future alignment or characterization techniques.

## 5. Conclusion   

NPG is an ideal 3D test object for characterizing TXM experimental setups operating at high photon energies. Different NPG samples with ligament sizes optimal for the expected spatial resolution at a specific TXM instrument can be manufactured and, following the robustness of the material itself, can even be transported to and scanned at different synchrotron-based or benchtop-based TXM instruments. Hereafter a TXM data repository based on scanned and analysed NPG samples can be used to compare different TXM instruments with each other, in terms of corrected horizontal and vertical drift, quality of the reconstructed data (before and after applying the joint re-projection method), as well as for 2D qualitative and 3D quantitative parameters and achievable 3D spatial resolution. The creation of such a proposed TXM data repository will also benefit from the usage of open source tools, such as the joint re-projection method, implemented in *TomoPy*, as well as by the ‘EM2EM/FSC’ functions in the *IMAGIC* image processing package and the *Pore3D* package for quantitative analysis.

## 6. Related literature   

The following reference, not cited in the main body of the paper, has been cited in the supporting information: Henke *et al.* (1993 ▸).

## Supplementary Material

Section S1: Calculation of the theoretically absorbed dose; and Figs. S1, S2 and S3.. DOI: 10.1107/S1600577518015242/pp5132sup1.pdf

Video S1: Video of projections of NPG (A) original and (B) after rapid alignment and forward projection (number of iterations: 40). The video shows every fifth projection of the entire scan.. DOI: 10.1107/S1600577518015242/pp5132sup2.avi

## Figures and Tables

**Table 1:** Extracted image descriptors (mean values and standard deviations) for both the air and Au parts, obtained by applying quantitative 3D image analysis on selected VOIs

	Quantitative 3D parameters	
	Air (porous part)	Au (solid part)
Morphological analysis		
Percentage volume (Vv) (%)	57.4 ± 0.3	42.6 ± 0.3
Specific surface area (Sv) (µm^−1^)	4.4 ± 0.1	4.4 ± 0.1

Anisotropic analysis		
Isotropy (–)	0.96 ± 0.00	0.96 ± 0.00
Elongation (–)	0.03 ± 0.00	0.03 ± 0.00

Skeleton analysis		
Connectivity density (Conn.D) (µm^−3^)	4.0 ± 0.4	0.6 ± 0.1

Blob analysis of inscribed blobs		
Node size (nm)	236 ± 83	241 ± 68
Node sphericity (–)	0.9 ± 0.1	1.0 ± 0.1
Throat size (nm)	175 ± 71	162 ± 65
Throat sphericity (–)	1.0 ± 0.1	1.0 ± 0.1
Maximum node size (nm)	352 ± 95	363 ± 75
Maximum node sphericity (–)	1.0 ± 0.1	1.0 ± 0.0
