# Supplementary material for: Nanoporous gold: a hierarchical and multiscale 3D test pattern for characterizing X-ray nano-tomography systems
Source: J Synchrotron Radiat. 2019 Jan 1;26(Pt 1):194–204. doi: 10.1107/S1600577518015242 (PMC6337890; doi:10.1107/S1600577518015242)
Supplement: Supplementary file 1 [file s-26-00194-sup1.pdf]

JOURNAL OF SYNCHROTRON RADIATION

Volume 26 (2019)

Supporting information for article:

Nanoporous gold: a hierarchical and multiscale 3D test pattern for characterizing X-ray nano-tomography systems

Emanuel Larsson, Doğa Gürsoy, Francesco De Carlo, Erica Lilleodden, Malte Storm, Fabian Wilde, Kaixiong Hu, Martin Müller and Imke Greving

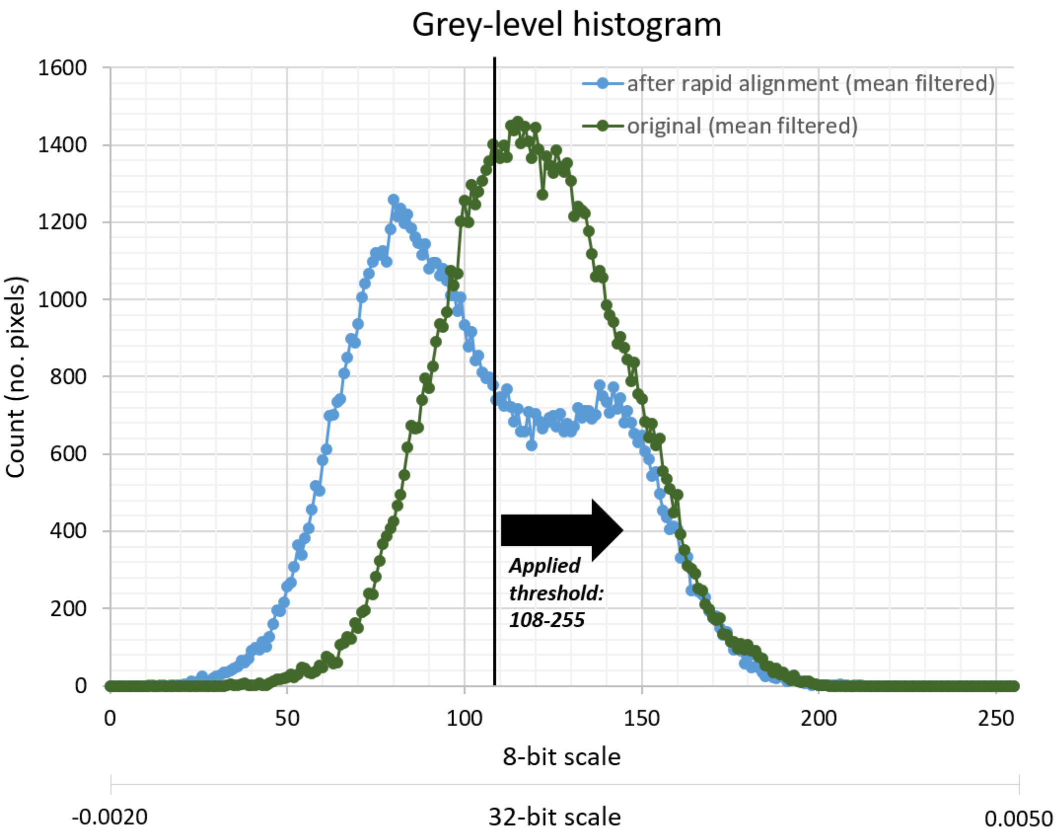

Figure S1 Grey level histogram of mean 3D mean filtered slices of both original (in dark green) and after rapid alignment of the projections (in blue). The histogram is displayed on both an 8-bit and 32-bit scale. (Please note that the application of the rapid alignment procedure of the projections leads to two distinct peaks in the corresponding reconstructed slice, which were separated using standard grey-level thresholding. For the original slice only one peak can be observed, which hampers consecutive segmentation procedures.)

Video S1 Video of projections of NPG A) original and B) after rapid alignment and forward projection (number of iterations: 40). The video shows every 5th projection of the entire scan.

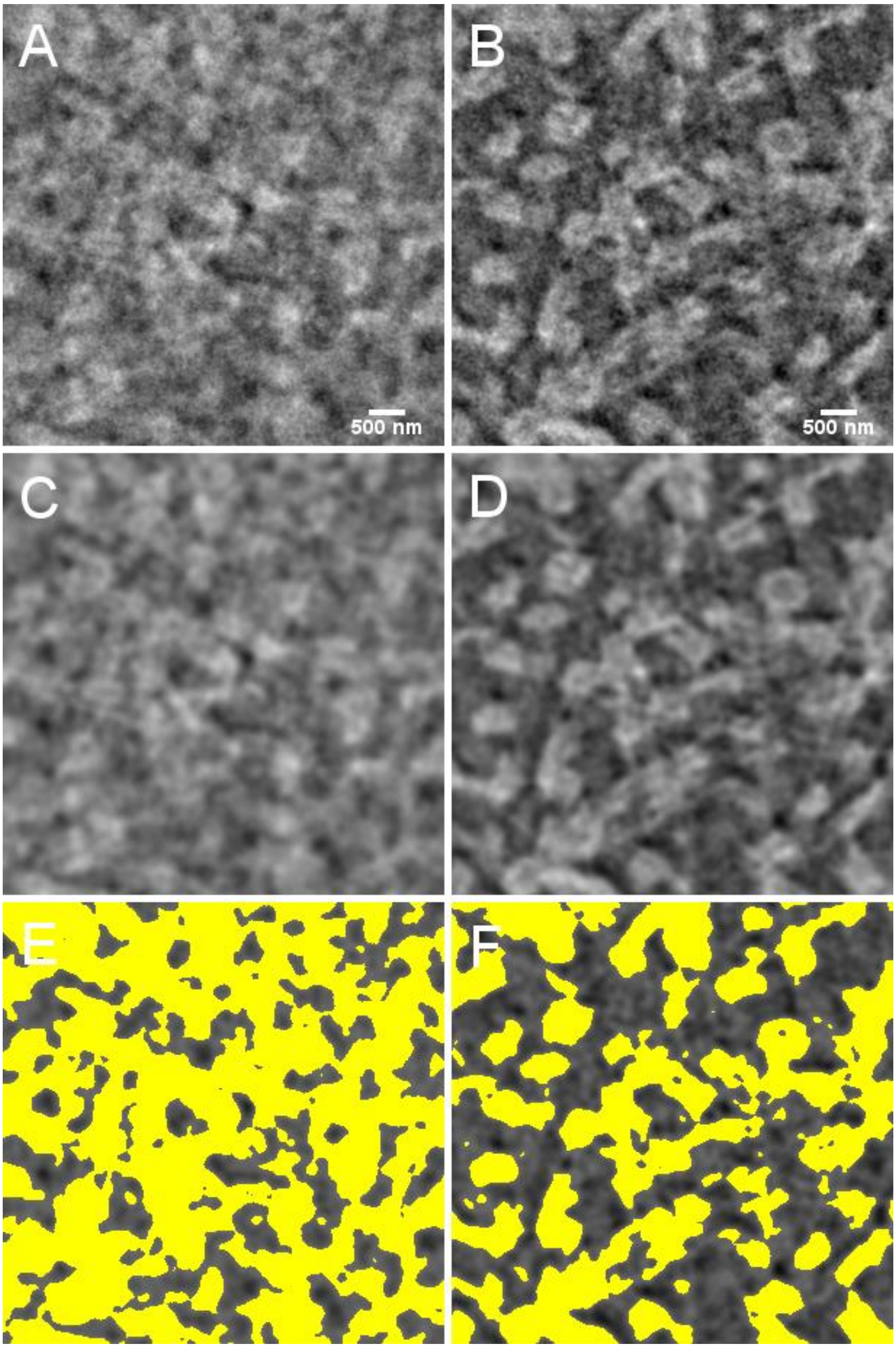

Figure S2 Selected Region of Interests (ROIs) (from Fig.5) of A) original and B) after rapid alignment of the projections followed by reconstruction with forward projection (number of iterations: 40). Corresponding 3D mean filtered slices are shown in C) and D) and segmented slices of the Au-part in E) and F) respectively. The images are displayed on an 8-bit grey-level scale.

S1. Calculation of the theoretically absorbed dose

The theoretically absorbed dose was calculated per single projection, as well as per entire tomographic scan of 450 projections, using equation 2:

$$D = \frac{\mu_{en}}{\rho} \cdot N \cdot E \cdot t \qquad (2)$$

where D is the dose in *Gray* or *Joules/m²*, $\frac{\mu_{en}}{\rho}$ is the mass energy absorption coefficient in m^2/kg of the absorbing medium, N is the photon fluence rate in $photon/m^2/s$, E is the photon energy in *Joules* and t is the acquisition time in *seconds*. Tabulated data from (Henke *et al.*, 1993) for the $\frac{\mu_{en}}{\rho}$ of Au, as well as measured fluency rates for eneriges between 10 to 16 keV at the P05 beam line at PETRA III were used to calculate the theoretically absorbed dose, as a function of the photon energy.

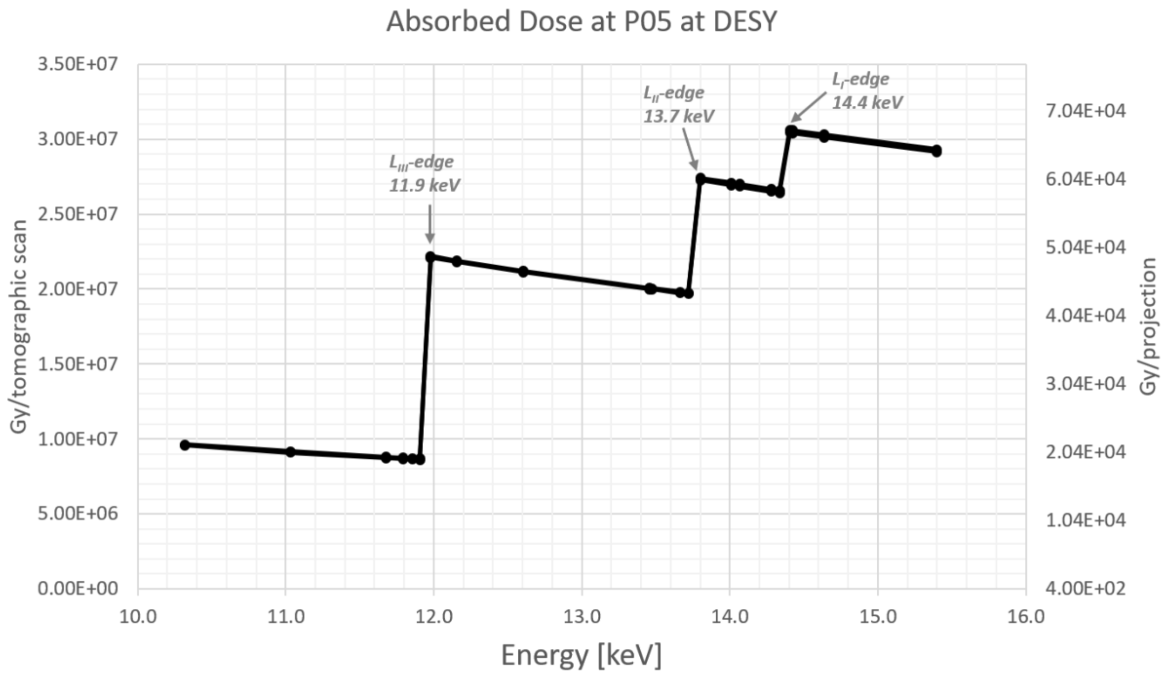

Figure S3 The theoretically absorbed dose for Au per projection and tomographic scan as a function of the photon energy. (Please note that the efficacy of the X-ray optics on the total absorbed dose has not been considered.)
